# 肺部结节（≤2 cm）楔形切除胸外科全国专家共识（2023版）

**DOI:** 10.3779/j.issn.1009-3419.2023.102.17

**Published:** 2023-05-20

**Authors:** HU Jian, CHEN Jun, CHEN Chang, ZHONG Wenzhao, GENG Qing, 肺部结节（≤2cm）楔形切除专家共识（2023版）编写委员会

**Affiliations:** ^1^310000 杭州，浙江大学医学院附属第一医院普胸外科; ^1^Department of Thoracic Surgery, the First Affiliated Hospital of Zhejiang University School of Medicine, Hangzhou 310000, China; ^2^310000 杭州，浙江省医疗器械临床评价技术研究重点实验室; ^2^Key Laboratory of Clinical Evaluation Technology for Medical Device of Zhejiang Province, Hangzhou 310000, China; ^3^300054 天津，天津医科大学总医院肺部肿瘤外科; ^3^Department of Lung Cancer Surgery, Tianjin Medical University General Hospital, Tianjin 300054, China; ^4^200433 上海，同济大学附属上海市肺科医院胸外科; ^4^Department of Thoracic Surgery, ShanghaiPulmonary Hospital, Tongji University, Shanghai 200433, China; ^5^519041 广州，广东省人民医院胸外科; ^5^Department of Thoracic Surgery, Guangdong General Hospital,Guangzhou 519041, China; ^6^430060 武汉，武汉大学人民医院胸外科; ^6^Department of Thoracic Surgery, Renmin Hospital of Wuhan University, Wuhan 430060, China

**Keywords:** 肺部结节, 楔形切除, 共识, Pulmonary nodules, Wedge resection, Consensus

## Abstract

肺癌是全球癌症相关死亡率最高的恶性肿瘤。早期非小细胞肺癌（non-small cell lung cancer, NSCLC）的治疗标准是根治性肺叶切除术，而近年来的各项研究发现肺部结节（≤2 cm）进行亚肺叶切除术不劣于标准肺叶切除术，甚至能改善患者预后。这些重要研究发现对于推进胸外科肺部结节（≤2 cm）的楔形切除形成行业共识与规范具有切实有效的积极意义。本共识的目的是提出肺部结节（≤2 cm）楔形切除在胸外科领域的全国专家共识。胸外科肺部结节（≤2 cm）楔形切除专家共识（2023版）编写委员会的各位专家共同参与了《肺部结节（≤2 cm）楔形切除胸外科全国专家共识（2023版）》的修订工作。专家们根据近年来国内外肺部结节（≤2 cm）楔形切除的临床研究进展，结合我国胸外科领域楔形切除的治疗规范，共同撰写了本次肺部结节（≤2 cm）楔形切除的全国专家共识。本共识从以下方面进行了整理：（1）肺部结节（≤2 cm）楔形切除的适应证；（2）肺部结节（≤2 cm）楔形切除的切除范围；（3）肺部结节（≤2 cm）适合楔形切除的方式。本次共识最终就8条推荐意见达成共识，并整理出5条尚存在争议、有待更多证据明确的意见，整合了全国胸外科各中心专家同道的意见，使得肺部结节（≤2 cm）的楔形切除更适应我国国情；使得肺部结节（≤2 cm）的楔形切除可以更加规范化、同质化地应用于临床。未来应基于我国肺癌诊治特点积累更多相关研究内容，以优化肺部结节（≤2 cm）的治疗决策。

## Abstract (en)

Lung cancer is the highest cancer-related mortality rate in the world, and is one of the most common malignancies. The standard treatment for early-stage non-small cell lung cancer (NSCLC) is radical lobectomy, while recent studies have found that sub-lobectomy of pulmonary nodules (≤2 cm) is not inferior to lobectomy and even improve the prognosis of the patients. These important findings will effectively and positively promote the formation of consensus and principles of wedge resection of pulmonary nodules (≤2 cm) in the field of thoracic surgery. The purpose of this study is to present a national expert consensus on wedge resection of pulmonary nodules (≤2 cm) in the field of thoracic surgery. The experts from Editorial Committee of Consensus on Wedge Resection of Lung Nodules (≤2 cm) (2023 Edition) jointly participated in the revision work. According to the clinical progress about the wedge resection of pulmonary nodules (≤2 cm) at home and abroad during recent years, experts jointly wrote Wedge Resection of Pulmonary Nodules (≤2 cm): a Consensus Statement by Specialists of Thoracic Surgery (2023 Edition), in combination with the homogeneous treatment principles of wedge resection in the field of thoracic surgery in China. This consensus was summarized from the following aspects: (1) Indications of wedge resection of pulmonary nodules (≤2 cm); (2) Resection range of pulmonary nodules (≤2 cm) required for wedge resection; (3) Excisable pulmonary nodules (≤2 cm) for wedge resection. This consensus finally put forward 8 recommended opinions, and sorted out 5 opinions which were still controversial and needed more evidence. The integrated opinions were generated through the discussion held among the experts of thoracic surgery from all over the country, making wedge resection of pulmonary nodules (≤2 cm) more appropriate for China and more standardized and homogeneous for clinical practice. In the future, more relevant researches should be accumulated based on the characteristics of lung cancer and its diagnosis and treatment in China, optimizing the treatment of pulmonary nodules (≤2 cm).

肺癌是全球癌症相关死亡率最高的恶性肿瘤^[1]^。早期非小细胞肺癌（non-small cell lung cancer, NSCLC）的标准治疗是根治性肺叶切除术，定义为切除出现癌变的肺叶，包括区域肺门和纵隔淋巴结^[2]^。而亚肺叶切除术包括了肺段切除（lung segmentectomy）和肺楔形切除（wedge resection of lung）。较早之前Ginsberg等^[3]^进行的一项随机对照研究比较了亚肺叶切除和肺叶切除在治疗结节直径≤3 cm的I期肺癌患者中的表现，但受限于当时入组的病例多为>2 cm的实性肿块且当时的手术器械较落后，所以该研究未能证明亚肺叶切除的非劣效性。同时，由于该研究对于肺部结节的直径分层分析不足，致使一段时间以来亚肺叶切除被认为是一种“妥协”的手术。然而，近年来的一些研究，诸如JCOG0802^[4]^、JCOG0804/WJOG4507L^[5]^等，对入组的肺部结节的直径、实性成分作了规定，如JCOG0802^[4]^中入组的肺部结节需≤2 cm且实性成分占比（consolidation tumor ratio, CTR）>0.5，而JCOG0804/WJOG4507L^[5]^中比较了≤2 cm且CTR≤0.25的肺部结节。两项研究结果的相继发表使≤2 cm的肺部结节的标准术式又重新成为了胸外科医师讨论的热点话题。作为重要的一种亚肺叶切除术，肺楔形切除术在肺癌的治疗中发挥着重要作用，具有手术操作及流程更加简化、患者创伤更小的明显优势^[6]^。同时随着肺部计算机断层扫描（computed tomography, CT）作为体检项目的普及，筛查出大量≤2 cm甚至是亚厘米的含磨玻璃成分的肺部结节，其中相当一部分为早期惰性肺癌。在目前的研究结果中此类结节更适合肺楔形切除术^[5,7]^。相较于肺段切除术及肺叶切除术，肺楔形切除术对患者术后肺功能影响更小，住院时间更短，术后生活质量更高^[8]^。但尽管肺楔形切除术简单易行，具有诸多优势，目前却缺少统一的手术标准及规范，迫切需要同质化。

为此，学组检索了PubMed、Web of Science等英文数据库以及万方、中国知网等中文数据库中关于肺部结节（≤2 cm）楔形切除的文献，选取其中证据级别较高、研究内容契合临床实践的文章，结合临床经验，制定相关问题，并且学组召开8次专家会议，逐步推进，最终在以下8条推荐意见达成共识，并整理出5条尚存在争议、有待更多证据明确的意见。

本共识推荐的级别为：

1A级：基于高水平证据[严谨的meta分析或随机对照试验（randomized controlled trial, RCT）结果]，专家组有统一认识。

1B级：基于高水平证据（严谨的meta分析或RCT结果），专家组有小争议。

2A级：基于低水平证据，专家组有统一认识。

2B级：基于低水平证据，专家组无统一认识，但争议不大。

3级：专家组存在较大争议。

## 1 肺部结节（≤2 cm）楔形切除的适应证

近年来随着低剂量螺旋CT体检筛查的普及，越来越多的肺部结节（≤2 cm）被发现，现在普遍认为其中相当一部分为惰性肿瘤^[9⇓-11]^。针对此类结节，首要的原则是正确把握手术介入时机，避免过度治疗。其次是避免错过治疗机会，以适当时机介入能够达到最微创的效果，切除范围最小，功能损伤最小，术后可以实现快速康复。

### 1.1 根治性肺楔形切除术

#### 1.1.1 影像学特征

胸部CT扫描发现的、以纯磨玻璃结节（ground glass opacity, GGO）为表现的早期肺癌，在临床、影像及病理特征方面具有高度一致性。出现实体成分的混合型GGO以及纯实性的肺部结节，在区分其良恶性方面则具有不同的异质性。对于肿瘤最大直径≤2 cm且CTR≤0.5（尤其是CTR≤0.25）的早期肺癌，其病理多为原位癌或微浸润癌，侵袭力极低，极少有胸膜、脉管浸润以及淋巴结转移^[7⇓-9]^。美国国立综合癌症网络（National Comprehensive Cancer Network, NCCN）指南^[8]^也指出此类肺部肿瘤可采用肺段或楔形切除^[12]^。对于这类肿瘤，行楔形切除的预后与行肺叶切除以及肺段切除间无明显差异。对此，JCOG0804/WJOG4507L试验^[5]^也给出了佐证，认为对于肿瘤直径<2 cm、CTR<0.25的肺癌患者，实施肺楔形切除可以达到与肺叶切除、肺段切除相同的预后。

除此之外，近年来一些学者对此也从不同角度提出了大致相近的看法。Im等^[13]^发现对于直径<3 cm的亚实性肿瘤，采取肺楔形切除的患者术后5年无复发生存（recurrence free survival, RFS）率与肺叶切除甚至肺段切除并无统计学差异。Cho团队^[14]^及Yano团队^[15]^均认为影像学非侵袭性（CTR≤0.25）可作为早期肺腺癌亚肺叶切除的一个较好的指标。王现国等^[16]^比较肺段切除术和肺楔形切除术对于直径≤2 cm且CTR≤0.5的肺腺癌治疗效果后发现，针对外周性（肺实质外1/3）且直径≤2 cm的早期肺腺癌，肺楔形切除优于肺段切除。这些观点无一例外地佐证了肺部结节（≤2 cm）可以采取肺楔形切除，以代替对肺损伤更大的肺段切除乃至于肺叶切除，达到统计学上无明显差异甚至更优的治疗效果。

推荐1：胸部CT发现的肺部<2 cm且CTR≤0.25的混合型GGO或纯GGO可选择肺楔形切除（2A）。

对于完全实性结节或短期随访后增大明显的结节，正电子发射计算机断层显像（positron emission tomography/CT, PET/CT）为推荐的筛查方案，可对恶性度高的肿瘤提前预警，为后续治疗争取时间。

推荐2：胸部CT发现的肺部实性高危结节或短期随访后进展的实性结节，PET/CT为可选的筛查方案（2A）。

#### 1.1.2 病理学特征

手术方式的选择与肺部结节的术前穿刺病理、术中冰冻病理以及术前科学随访密切相关，应根据不同的情况选择合适的手术方式^[17]^。术前穿刺病理对于周围型小结节来说尚有争议，虽然它对明确病理诊断有帮助，但也存在一定的误诊率和漏诊率^[18]^。对于可疑转移淋巴结，可通过超声引导下经支气管镜针吸活检（endobronchial ultrasound-guided transbronchial needle aspiration, EBUS-TBNA），以达到准确分期，为手术方案选择提供依据^[19]^。除上述创伤性诊断手段之外，也可以通过CT、PET/CT等非创伤性手段，获取肺结节的良恶性倾向^[20]^。

推荐3：对于术前尚未明确是否为肺部恶性肿瘤的病灶，可以行楔形切除的结节优先选择楔形切除，待术中病理报告决定是否进一步手术（2A）。对于术中冰冻病理证实为良性的肺部结节，实施肺楔形切除即可（1A）。

对于肺部恶性结节，病理学大致可分为鳞癌、腺癌、腺鳞癌、神经内分泌肿瘤、大细胞癌、肉瘤样癌、其他上皮源性肿瘤、转移性肿瘤等^[12]^。按照恶性程度、临床特点、治疗方式及预后不同，原发性肺癌主要分为NSCLC、小细胞肺癌（small cell lung cancer, SCLC）两大类^[12]^。

NCCN指南认为病理为原位腺癌（adenocarcinoma in situ, AIS）的<2 cm的周围型肺结节可采用肺楔形切除术^[12]^。除此之外，国内《中华医学会肺癌临床诊疗指南（2022版）》将直径≤2 cm的周围型小结节且术中冰冻病理为非浸润性肺癌，即不典型腺瘤样增生（atypical adenomatous hyperplasia, AAH）、AIS或微浸润性腺癌（minimally invasive adenocarcinoma, MIA），均作为意向性楔形切除的首选推荐类型^[21]^。

病理学上的非侵袭性肺部肿瘤指的是无淋巴结转移或脉管浸润，如AAH、原位癌、微浸润性癌（腺癌、鳞癌）等。影像学上非侵袭性肺部肿瘤最初指的是CTR<0.5的肺部结节，后来认为对于预测肺部肿瘤是否具有侵袭性，CTR<0.25可能是更好的截断值^[22]^。对此，Yoshida等^[23]^表示，薄层CT中的肺部肿瘤大小及CTR均可反映其是否易出现侵袭性而影响亚肺叶手术的预后。

肺部结节行楔形切除是否需要做淋巴结清扫，目前仍存在争议。相对于肺段、肺叶切除术，肺楔形切除术无法获得肺叶内淋巴结（12组及以上）。但对于肿瘤最大直径≤2 cm且CTR≤0.5（尤其是CTR≤0.25）的早期肺癌，极少出现淋巴结转移^[7⇓-9]^。

推荐4：肺部结节（≤2 cm）术中冰冻病理为非侵袭性肺部肿瘤，如微浸润性癌，可选择肺楔形切除术及伴或不伴淋巴结采样（1A）。

对于侵袭性癌，目前术式选择仍有一定争议，详见附录。

### 1.2 姑息性肺楔形切除术

肺楔形切除术在保留患者术后肺功能方面具有较明显优势。相比于肺叶切除术，肺楔形切除术更少地切除肺实质，在保留术后肺功能尤其是第一秒用力呼气容积（forced expiratory volume in the first second, FEV_1_）方面具有优势^[24,25]^。Mahesh团队^[26]^认为对于心肺功能储备不良的患者，肺楔形切除是一种安全可行的手术方式。但部分患者肺功能检查结果不准确，存在配合不佳、门齿漏风等因素干扰，因此，血气分析、运动试验、闭气试验等也是肺功能情况评判的重要依据。另外，部分患者因高龄、有其他较严重的伴随疾病等手术高风险因素存在，在综合评估后，可选择姑息性肺楔形切除术，以提高手术安全性。

同时，部分患者肺门区存在严重的门钉钙化淋巴结侵袭肺血管、支气管，行肺段/肺叶切除术难度极大，大大增加出血、支气管胸膜瘘等严重并发症发生风险。综合评估后，可考虑姑息性肺楔形切除术。

部分患者不能耐受肺段/肺叶切除术，或患者肿瘤已进展至中晚期无法根治切除，且解剖位置特殊，难以穿刺获得病理，或穿刺多次无法获得阳性结果，为获取病理学诊断，可根据实际情况选择姑息性肺楔形切除术。

推荐5：患者全身状况无法耐受肺叶切除术或手术风险过高，经综合评估后，可选择姑息性肺楔形切除术，术后辅以其他非手术治疗方案（2A）。肿瘤已进展至中晚期无法根治切除，可选择肺楔形切除术作为获取病理学诊断的一种候选方案（2A）。

### 1.3 多发肺部结节（≥3枚）的手术选择

多发肺部结节（≥3枚）在结合患者病史排除转移后，应重点关注主病灶（或称为高危病灶），即综合术前及术中评估，在同一患者的肺部多个病灶中体积较大、分期较晚的病灶^[27,28]^。多发肺部结节手术选择应谨慎，若多发肺部结节无法完全切除，原则上应以切除高危病灶为主要目标，尽可能保留肺功能，以获得肺部结节的病理及分子诊断，有利于制定下一步治疗方案^[29]^。

对于可完全切除的多发肺部结节，亚肺叶切除是比较理想的选择，对比肺叶切除，预后相似同时可保留更好的肺功能^[29]^。如多发病灶位于同侧胸腔内，在可保证患者生活质量的前提下，考虑切除多处高危病灶。若多发病灶位于双侧胸腔，则可根据患者全身情况及心肺功能选择仅切除单侧胸腔高危结节，或者切除同期双侧胸腔高危结节，或者切除分期双侧高危结节^[30]^。

推荐6：多发肺部结节手术选择应谨慎，原则上应以切除高危病灶为主要目标，尽可能保留肺功能，优势部位的楔形切除为最优选择。获得肺部结节的病理及分子诊断有利于制定下一步治疗方案（2B）。

## 2 肺部结节（≤2 cm）楔形切除所需的切除范围

根据之前的研究，当≤2 cm的病灶被切除时，如果能获得足够的无瘤边缘，对于I期NSCLC来说，亚肺叶切除术在肿瘤学上可能等同于肺叶切除术^[31]^。Sawabata等^[32]^报道，对于NSCLC，如果肿瘤与切除线之间的距离大于肿瘤自身的最大直径，则可获得无瘤边缘。在Mohiuddin等^[33]^关于≤2 cm的NSCLC的研究中，1.5 cm的距离被认为是产生肿瘤浸润边缘的边界，而无论肿瘤大小，2 cm或更大的距离通常与无瘤边缘相关。此外，Sato等^[34]^也认为肺部结节楔形切除的切缘应大于肿瘤最大直径或至少2 cm。《中华医学会肺癌临床诊疗指南（2022版）》建议：影像学上以GGO表现为主的≤2 cm的肺结节，楔形切除应保证肉眼可见切缘>5 mm，若≤5 mm，需冰冻切片证实切缘阴性；不要求常规行淋巴结活检；若遇到明显肿大淋巴结，则需采样^[17]^。

推荐7：纯GGO肺部结节（≤2 cm）楔形切除冰冻切片证实切缘阴性，无瘤切缘应至少>5 mm（2A）。含实性成分肺部结节（≤2 cm）楔形切除冰冻切片证实切缘阴性，无瘤切缘应大于肿瘤最大直径（2A）。

## 3 肺部结节（≤2 cm）适合楔形切除的方式

### 3.1 可行楔形切除的肺部结节（≤2 cm）的位置

Landreneau等^[35]^认为，肺楔形切除适用于结节位于肺外周1/3带的患者。但若肺楔形切除术中损伤或压迫肺段级及以上的血管或支气管，则该区域的肺组织功能将极大受损，更适宜肺段或肺叶切除术。考虑到切割缝合器的宽度，根据切缘的要求，肺结节应满足距离肺段及以上血管、支气管一定的安全距离，同时结节的位置应距肺表面小于该方向上肺门至肺表面距离的1/3（即肺野外1/3带），此时实施肺楔形切除术则较为安全^[36]^。另外，结节生长在双肺尖段肺尖、前段靠前、肺裂边、背段尖、基底段裙边等较易使用肺钳及吻合器的位置，我们认为，这也是肺楔形切除术的可选位置。除此之外，如果结节生长在下肺韧带边、发育不佳的肺裂附近，通过一定的解剖性分离也可达到的位置，实施肺楔形切除术也较为容易。

### 3.2 肺部结节（≤2 cm）楔形切除的优势解剖位置

对于贴近胸膜生长的肺部结节（距离胸膜<1 cm），肺楔形切除术具有天然优势，可优先选择肺楔形切除术。另外距离成熟肺边界（前缘、后缘、肺尖部、基底段裙边）<2 cm，或距离成熟（或通过分离达到）肺裂边界<2 cm，同样可优先选择肺楔形切除术。这些区域的结节行楔形切除术不仅可以简化手术步骤，同时相对其他位置的肺楔形切除术，还可以尽量减少手术对于正常肺实质的损耗，患者术后康复更快，术后疼痛更轻，并发症发生率也更低，部分患者还可选择无管手术、日间手术^[37]^。

### 3.3 肺部结节（≤2 cm）楔形切除的推荐方式

现今肺楔形切除术中术者多使用切割缝合器。切割缝合器的广泛使用，使肺部手术的安全性大为提高，同时减少了术中出血，使切口微创化，实现了快速康复^[38]^。肺楔形切除术目前没有标准的切除步骤和规范，固然想象中相对简单，但不同主刀医生对肺楔形切除术的实际操作有相当差异，使得该术式相比肺叶切除术、肺段切除术更难做到同质化。对患者而言，不规范的肺楔形切除术可能导致正常肺部组织切除过多而不必要地损失肺功能，或切除范围不够而提高了肿瘤复发风险，甚至切缘肿瘤残留^[39,40]^。肺楔形切除术的规范化、同质化是对广大患者的更好保障，同时也在一定程度上减少了术者的失误率。

对于原发于肺表面的肺部结节，术者在术中持械操作时应避免直接夹持到结节所在区域，以减少肿瘤胸腔播散的风险。在实施肺楔形切除术中，尽量按支气管走行区域切除肺部组织，利于切除后残肺的舒张，减少残肺的扭转，减少肺通气血流比的失衡区域，保留更多肺功能，减少术后并发症^[41]^。同时应避免夹取过多肺组织导致切割缝合器钉仓闭合欠佳而造成肺实质漏气、渗血、非计划二次手术及术后咯血等不必要的并发症，或因钉仓闭合太紧密造成肺组织皱缩而影响肺复张^[42]^。

3.3.1 优势位置肺部结节（≤2 cm）楔形切除的推荐方式 对于优势楔形切除位置的肺部结节，距离胸膜1 cm以内的，建议待肺塌陷理想后，夹持结节附近组织提起，以顺着该区域支气管走形方向，以适当切缘切除结节。距离肺边界或肺裂边界<2 cm的肺部结节，建议夹持肺边拉长，以顺边界的方向，以适当切缘切除结节。

3.3.2 非优势位置肺部结节（≤2 cm）楔形切除的推荐方式 对于非优势位置的肺部结节，选择肺楔形切除时应在保证切缘的前提下，充分利用周围的肺部解剖结构，保留更多肺功能。结节距离肺边界或肺裂边界较近的，可顺边界方向提起，切除足够范围的肺组织。结节距离肺边界或肺裂边界较远的，应夹持肺部组织尽量提起，塑形，顺支气管走形方向切除。结节位于纵隔面、橫膈面的结节，可尝试提起附近组织切除；如结节附近肺组织提起困难，应利用肺边界或肺裂边界切除。非优势位置的肺楔形切除，应充分做好术前规划，如有条件，可依据三维重建等技术支持，更有利于患者术后快速恢复。

### 3.4 肺部结节（≤2 cm）的术前定位

对于选择肺楔形切除的肺部结节，需要术前精确定位，以确保手术时定位准确且切缘足够。若穿刺针不慎脱钩，可及时标记肺部结节周围，保障安全切缘。术前精确定位适用于结节位置因距开孔位置较深而不宜触及，或位于肺表面以下较深位置、非隆起状或与周围正常肺实质颜色相近不易辨别的肺结节^[18,43,44]^。

据报道^[45]^，混合型GGO的触诊成功率为75%（15/20），而纯GGO仅为12.1%（4/33）。因此，手指定位或许应仅作为一项补充技术，或者考虑使用术中解剖定位法，为肺部结节实行楔形切除创造必要条件^[43,44]^。

此外，对于手术中难以触及、缺少解剖定位标记的肺部结节，若需要做楔形切除，应充分做好术前手术规划，特别是可以选择术前CT引导下留置定位装置，如Hook-wire定位针、定位弹簧圈、定位钩等，引导术中予以该区域的精准切除^[18,43,44]^。

### 3.5 不宜行楔形切除的肺部结节（≤2 cm）

对于胸部CT发现的<2 cm且CTR<0.5的混合型GGO或纯GGO，但结节位置不在楔形可切除范围内的，为保留更多肺组织、减少肺功能损失，肺段切除术、亚段切除术、联合亚段切除术不失为一种较好的选择。

推荐8：手术以无瘤原则为前提（参照推荐7），肺楔形切除应尽量缩小切除范围，保障患者术后肺功能的恢复（2A）。对于难以实现肺楔形切除术的小结节（≤2 cm），仍建议采用肺段、亚段、联合亚段、联合肺段切除术（2A）。

## 4 小结

本共识是国内第一个聚焦于肺部结节（≤2 cm）楔形切除的专家共识。关于肺部结节（≤2 cm）的亚肺叶切除一直是胸外科领域的研究热点，但大多数专家学者关注于肺段切除与肺叶切除的差异比较，临床中应用更为基础的楔形切除尚存在一些争议。基于此现状，我们在充分检索文献的基础上，组织相关专家进行讨论，结合我国实际情况，共同制定了本共识，以期为胸外科肺部结节（≤2 cm）的楔形切除提供决策依据，规范手术标准，为患者带来同质化治疗结局。对于目前研究少、证据不足、存在争议、难以形成共识的内容，我们在文章的最后添加了一份附录，为今后相关临床研究的开展提供方向和目标，留待以后进一步的研究来统一观点。

需要声明的是，本共识是基于目前检索可得到的文献资料以及参与讨论的专家所掌握的循证医学证据制定，仅供临床医护人员参考应用，不作为任何医疗纠纷及诉讼的法律依据。

**附录：**以下为目前存在争议、临床研究开展不足或者尚未开展的内容，尚处于探索中，希望为以后临床研究的开展和共识的书写提供方向和目标。

1. 对于术中冰冻病理报告为浸润性腺癌的肺部结节（≤2 cm）且CTR≤0.5，应采用的手术方式仍存在争议，包括肺叶切除术、肺段切除术及肺楔形切除术。根据以往的经验，浸润性腺癌建议行肺叶切除术，以降低复发率。但根据全球相关前瞻性研究结果，对于符合影像学要求的浸润性腺癌，行包括肺楔形切除在内的亚肺叶切除术即可^[22]^。同时术中冰冻病理报告的准确性与病理制片、切片部位、病理医生的经验也有一定相关性。同时该类结节手术纵隔淋巴结是否需要清扫，也存在一定争议。故浸润性腺癌的手术方式选择仍待更多临床研究及数据分析提供支持。

2. 肺部结节（≤2 cm）病理为NSCLC且包含微乳头、实性亚型、黏液、复杂腺体结构、低分化或未分化成分等复发高危因素，不建议行肺楔形切除术。NSCLC主病灶周围存在脉管癌栓、胸膜侵犯、微卫星转移灶、气腔播散等肿瘤分期升级情况，不建议行肺楔形切除术。但目前因病理科技术的限制以及各地病理科经验的差异，术中冰冻很难准确报出危险亚型。国内学者发起的一项临床研究^[46]^表明术中病理区分高危亚型是可行的。另外如术中冰冻报告中有分化程度提示，对术式选择也有一定的帮助，有待病理学同道的进一步推动改进。

3. 病理类型为SCLC的肺部结节（≤2 cm），根据目前NCCN SCLC诊疗规范推荐，建议行前哨淋巴结活检，如淋巴结没有明显转移（I期-IIA期），仍建议行肺叶切除。如淋巴结存在转移（IIB期以上），治疗方案存在争议，可选择姑息性肺楔形切除术或肺叶切除术。可根据该争议做更多研究明确。

4. 肺部结节的CT值，作为反映其实际内在构成的密度指标，对于胸外科医师判断其病理性质可能有重要参考价值，也是肺部结节手术方式选择的重要参考指标之一。但因CT机器的型号、规格、扫描层厚、患者体位、结节内渗血等，均可对其测量结果造成影响。因此，目前尚无研究明确得出关于准确预测肺部结节病理性质的CT值阈值的报告。针对CT值阈值是否在辅助判断肺部结节病理性质方面具有价值，尚待更多研究发表支持。

5. 目前肺楔形切除的微创手术方式，由于切口位置、手术器械的限制以及术者经验的影响，存在一定比例的楔形切除难以达到理想的标准切缘的病例，即在尽可能保留肺功能的同时，又尽可能降低复发率。在人工智能快速发展的时代，研发肺楔形切除的专病机器人正在成为可能。通过机器人的辅助，术者可以在胸腔内完成结节的定位、切除范围规划、肺部结节切除等，对未来肺楔形切除术手术做到精准、快捷、经济及治疗效果同质化具有十分重要的意义。

**备注：**肺楔形切除地图（肺楔切地图）：为了更直观、立体、通透地显示适合进行肺楔形切除的优势区域，通过研究优势楔形切除区域的定义并结合临床实践经验，在三维重建技术的帮助下，制定了肺楔形切除地图，旨在使肺楔形切除更为规范化和同质化。肺楔形切除地图可以指导并确认肺楔形切除的优势部位，从而在根治性切除早期病灶的基础上减轻手术创伤、缩短手术时间、保留更多的肺组织，并为今后研发肺结节专病手术智能机器人奠定基础。

肺部结节（≤2 cm）楔形切除专家共识（2023版）编写委员会

**主编**

胡坚（浙江大学医学院附属第一医院）、陈军（天津医科大学总医院）、陈昶（同济大学附属上海市肺科医院）、钟文昭（广东省人民医院）、耿庆（武汉大学人民医院）

**执笔专家**

程钧（浙江大学医学院附属第一医院）、马少华（北京大学肿瘤医院）、汪路明（浙江大学医学院附属第一医院）、蒲强（四川大学华西医院）、李树本（广州医科大学附属第一医院）

**共同执笔专家**

吴楠（北京大学肿瘤医院）、龙浩（中山大学肿瘤防治中心）、刘德若（中日友好医院）、彭忠民（山东第一医科大学附属省立医院）、何哲浩（浙江大学医学院附属第一医院）

**撰写小组专家**（按姓氏汉语拼音排序）

蔡开灿（南方医科大学南方医院）、曾剑（中国科学院大学附属肿瘤医院）、陈铭伍（广西医科大学第一附属医院）、范庆浩（金华市人民医院）、冯靖祎（浙江大学医学院附属第一医院）、郭占林（内蒙古医科大学附属医院）、黄日胜（温州市中心医院）、姜杰（厦门大学附属第一医院）、乐涵波（舟山医院）、李晨蔚（宁波大学附属第一医院）、李单青（北京协和医院）、李鹤成（上海交通大学医学院附属瑞金医院）、李强（四川省肿瘤医院）、李向楠（郑州大学第一附属医院）、梁朝阳（中日友好医院）、林勇斌（中山大学肿瘤防治中心）、马金山（新疆维吾尔自治区人民医院）、沈琦斌（湖州市中心医院）、沈韦羽（宁波市医疗中心李惠利医院）、孙大强（天津市胸科医院）、孙诠（山西省肿瘤医院）、孙伟（海南医学院第二附属医院）、谭锋维（中国医学科学院肿瘤医院）、王海涛（浙江省人民医院）、王述民（北部战区总医院）、谢德耀（温州医科大学附属第一医院）、徐文震（三门县人民医院）、许顺（中国医科大学附属第一医院）、许志扬（莆田市第一医院）、杨帆（北京大学人民医院）、杨刚（铜陵市立医院）、叶波（浙江大学医学院附属杭州市胸科医院）、喻本桐（南昌大学第一附属医院）、喻光懋（绍兴市人民医院）、张春芳（中南大学湘雅医院）、张军（嘉兴市第二医院）、张毅（首都医科大学宣武医院）、赵纯（丽水市中心医院）、赵国芳（中国科学院大学宁波华美医院）、郑斌（福建医科大学附属协和医院）、郑勇洪（浙江衢化医院）、周仕宏（浙江大学医学院附属第一医院）

**审核小组专家**（按姓氏汉语拼音排序）

曹庆东（中山大学附属第五医院）、陈保富（台州市中心医院）、陈椿（福建医科大学附属协和医院）、陈锋夏（海南省人民医院）、陈海泉（复旦大学附属肿瘤医院）、陈和忠（海军军医大学第一附属医院）、陈剑锋（福建医科大学附属第一医院）、陈克能（北京大学肿瘤医院）、陈奇勋（中国科学院大学附属肿瘤医院）、陈秋强（湖州市第一人民医院）、陈志军（舟山医院）、崔永（北京友谊医院）、丁建勇（复旦大学附属中山医院）、董礼文（杭州市中医院）、范江（上海市第一人民医院）、方文涛（上海交通大学医学院附属胸科医院）、付军科（西安交通大学医学院第一附属医院）、付向宁（华中科技大学同济医学院附属同济医院）、葛棣（复旦大学附属中山医院）、耿国军（厦门大学附属第一医院）、谷志涛（上海市胸科医院）、顾春东（大连医科大学附属第一医院）、韩开宝（厦门弘爱医院）、韩育宁（宁夏医科大学总医院）、何建行（广州医科大学附属第一医院）、何正富（浙江大学医学院附属邵逸夫医院）、贺靳贤（宁波市医疗中心李惠利医院）、黄云超（云南省肿瘤医院）、江洪（杭州市第一人民医院）、姜涛（空军军医大学唐都医院）、蒋伟（复旦大学附属中山医院）、矫文捷（青岛大学附属医院）、康明强（福建医科大学附属协和医院）、雷杰（空军军医大学唐都医院）、李高峰（云南省肿瘤医院）、李小飞（西安国际医学中心医院胸科医院）、李印（中国医学科学院肿瘤医院）、李忠诚（青海大学附属医院）、梁志刚（宁波大学附属第一医院）、廖永德（华中科技大学同济医学院附属协和医院）、林一丹（四川大学华西医院）、刘宝东（首都医科大学宣武医院）、刘高峰（解放军联勤保障部队第988医院）、刘宏旭（辽宁省肿瘤医院）、刘建阳（吉林省肿瘤医院）、刘俊峰（河北医科大学第四医院）、刘彦国（北京大学人民医院）、刘阳（中国人民解放军总医院第一医学中心）、鲁继斌（中国医科大学附属盛京医院）、罗清泉（上海交通大学医学院附属胸科医院）、闾夏轶（浙江大学医学院附属第一医院）、吕望（浙江大学医学院附属第一医院）、马德华（浙江省台州医院）、马冬春（安徽省胸科医院）、马海涛（苏州大学附属第一医院）、茅乃权（广西医科大学附属肿瘤医院）、梅宏（贵州省人民医院）、梅建东（四川大学华西医院）、梅新宇（中国科学技术大学附属第一医院）、孟龙（山东省立医院）、牟巨伟（中国医学科学院肿瘤医院）、尼平（西藏自治区人民医院）、彭笑怒（烟台毓璜顶医院）、钱有辉（深圳大学第一附属医院）、乔贵宾（广东省人民医院）、曲昌发（哈尔滨医科大学附属肿瘤医院）、孙艺华（复旦大学附属肿瘤医院）、谭黎杰（复旦大学附属中山医院）、谭群友（陆军军医大学第三附属医院）、田辉（山东大学齐鲁医院）、王博（武汉大学人民医院）、王光锁（深圳市人民医院）、王海东（陆军军医大学第一附属医院）、王继勇（广州中医药大学第一附属医院）、王明松（上海交通大学医学院附属第九人民医院）、王群（复旦大学附属中山医院）、王正（广东省深圳市人民医院）、魏立（河南省人民医院）、吴丹（慈溪市人民医院）、吴庆琛（重庆医科大学附属第一医院）、吴中杰（嘉兴市第一医院）、冼磊（广西医科大学第二附属医院）、徐步远（平阳县人民医院）、徐侃（杭州市红十字会医院）、徐美青（中国科学技术大学附属第一医院）、徐全（江西省人民医院）、徐然（中国医科大学附属盛京医院）、许林（江苏省肿瘤医院）、许世广（北部战区总医院）、薛磊（海军军医大学第二附属医院）、薛涛（东南大学附属中大医院）、闫小龙（空军军医大学唐都医院）、杨劼（佛山市第一人民医院）、杨金良（河北医科大学第三医院）、杨明磊（中国科学院大学宁波华美医院）、杨异（上海市第六人民医院）、姚烽（上海市胸科医院）、叶敏华（浙江省台州医院）、于振涛（中国医学科学院肿瘤医院深圳医院）、张翀（浙江大学医学院附属第一医院）、张兰军（中山大学附属肿瘤医院）、张力为（新疆医科大学第一附属医院）、张临友（哈尔滨医科大学附属第二医院）、张苏宁（中国医科大学附属盛京医院）、张真榕（中日友好医院）、张志豪（中国人民武装警察部队海警总队医院）、赵光强（云南省肿瘤医院）、赵珩（上海市胸科医院）、赵晋波（空军军医大学唐都医院）、赵军（苏州大学附属第一医院）、赵松（郑州大学第一附属医院）、朱成楚（温州医科大学附属台州医院）、朱有才（浙江省荣军医院）、朱余明（同济大学附属上海市肺科医院）、祝鑫海（浙江医院）

**学会**

中国医疗保健国际交流促进会胸外科分会、中国医药教育协会胸外科专业委员会、海峡两岸医药卫生交流协会胸外科专业委员会、浙江省医学会胸外科学分会主任委员、浙江省医师协会胸外科医师分会、浙江省预防医学会肺癌预防与控制专委会
